# Patient blood management (PBM) in pregnancy and childbirth: literature review and expert opinion

**DOI:** 10.1007/s00404-019-05374-8

**Published:** 2019-11-14

**Authors:** Daniel Surbek, Yvan Vial, Thierry Girard, Christian Breymann, Gabriela Amstad Bencaiova, David Baud, René Hornung, Behrouz Mansouri Taleghani, Irene Hösli

**Affiliations:** 1 Department of Obstetrics and Gynaecology, Bern University Hospital, Insel Hospital, University of Bern, Friedbühlstrasse 19, 3010 Bern, Switzerland; 2 Clinic of Obstetrics and Gynaecology, University Hospital Basel, Basel, Switzerland; 3 Service of Obstetrics, Department Woman-Mother–Child, University Hospital (CHUV) and University of Lausanne (UNIL), Lausanne, Switzerland; 4 Department of Anaesthesiology, University Hospital Basel, Basel, Switzerland; 5 Obstetric Research-Feto Maternal Haematology Unit, University Hospital Zurich, Zurich, Switzerland; 6 Department of Obstetrics and Gynaecology, St. Gallen Cantonal Hospital, St. Gallen, Switzerland; 7 Department of Haematology, Inselspital, University and University Hospital Bern, Bern, Switzerland

**Keywords:** Patient blood management, PBM, Outcome, Improve, Perinatal haemorrhage, PPH, Pregnancy, Obstetrics

## Abstract

**Purpose:**

Patient blood management [PBM] has been acknowledged and successfully introduced in a wide range of medical specialities, where blood transfusions are an important issue, including anaesthesiology, orthopaedic surgery, cardiac surgery, or traumatology. Although pregnancy and obstetrics have been recognized as a major field of potential haemorrhage and necessity of blood transfusions, there is still little awareness among obstetricians regarding the importance of PBM in this area. This review, therefore, summarizes the importance of PBM in obstetrics and the current evidence on this topic.

**Method:**

We review the current literature and summarize the current evidence of PBM in pregnant women and postpartum with a focus on postpartum haemorrhage (PPH) using PubMed as literature source. The literature was reviewed and analysed and conclusions were made by the Swiss PBM in obstetrics working group of experts in a consensus meeting.

**Results:**

PBM comprises a series of measures to maintain an adequate haemoglobin level, improve haemostasis and reduce bleeding, aiming to improve patient outcomes. Despite the fact that the WHO has recommended PBM early 2010, the majority of hospitals are in need of guidelines to apply PBM in daily practice. PBM demonstrated a reduction in morbidity, mortality, and costs for patients undergoing surgery or medical interventions with a high bleeding potential. All pregnant women have a significant risk for PPH. Risk factors do exist; however, 60% of women who experience PPH do not have a pre-existing risk factor. Patient blood management in obstetrics must, therefore, not only be focused on women with identified risk factor for PPH, but on all pregnant women. Due to the risk of PPH, which is inherent to every pregnancy, PBM is of particular importance in obstetrics. Although so far, there is no clear guideline how to implement PBM in obstetrics, there are some simple, effective measures to reduce anaemia and the necessity of transfusions in women giving birth and thereby improving clinical outcome and avoiding complications.

**Conclusion:**

PBM in obstetrics is based on three main pillars: diagnostic and/or therapeutic interventions during pregnancy, during delivery and in the postpartum phase. These three main pillars should be kept in mind by all professionals taking care of pregnant women, including obstetricians, general practitioners, midwifes, and anaesthesiologists, to improve pregnancy outcome and optimize resources.

## Introduction

Patient blood management (PBM) has recently been introduced in several areas in medicine. It comprises a series of measures and methods to maintain an optimal haemoglobin (Hb) level, optimise haemostasis, minimise blood loss, and limit blood transfusions aiming to improve patient outcomes. Recent studies have shown that the use of PBM minimizes perioperative bleeding, reduces the requirement of blood transfusion [1–8], perioperative morbidity [1–4], mortality, duration of hospitalization [1, 4], and costs [9, 10]. Regarding this, the World Health Organization (WHO) was recommending since 2010 to urgently implement PBM [11]. Some hospitals have already successfully implemented PBM notably in Australia [7], Europe [2, 4–6], United States [3], and also in Asia. However, many obstetricians and obstetrical departments still are in need of guidance for the implementation of PBM in daily clinical practice. Despite the demonstrated benefits, challenges and mindsets limit the implementation of PBM guidelines into daily medical practice [12]. These challenges can be due to ignorance, lack of cross-functional engagement, limited resources, and miss conception of PBM benefits, which may even create a fear of job loss in blood donor service or transfusion medicine.

Introducing PBM in a hospital requires that the involved physicians and health care providers give up the sometimes-dogmatic idea that blood transfusions are the first line treatment to correct low haemoglobin values. PBM is based on three pillars (Table 1): (1) anticipation, detection, and correction of preoperative/prepartum anaemia; (2) prevention and reduction of perioperative/peripartum red blood cell (RBC) loss; and (3) optimizing postoperative/postpartum treatment of anaemia, including the restrictive use of red blood cell transfusions. Probably, the most difficult task is to convince hospital leaders of the importance of PBM and to change the culture of medical management among colleagues.

**Table 1:** PBM in obstetrics requires medical and surgical/interventional strategies for prevention and treatment of PPH

Medical strategies	Surgical/interventional strategies
Pre-partum Correction of anaemia Intra-partum Uterotonics [PPH prevention and treatment] Active coagulation management in PPH (Tranexamic acid, Fibrinogen, FFP) Post-partum Correction of anaemia Avoid unnecessary blood transfusions	Surgical technique minimizing blood loss (prevention of PPH) Balloon catheter Compression sutures Embolisation Cell salvage and autologous transfusion

## Method

With this literature review, we aimed to identify: (a) the patient at risk of PPH; (b) the need of PBM in obstetrics; and (c) PBM programs implementation in the field of obstetrics. The rationale for this literature review was to identify gaps in knowledge and to provide recommendations for the improvement of antenatal care for health care professionals in this crucial and fast evolving field of patient blood management.

Databases searched included PubMed, Scopus, CINAHL, Google, WHO guidelines database, Cochrane Library, and Web of Science, using search terms: PBM, PBM in obstetrics, blood transfusion, volume management, anaemia in pregnancy, and PPH. In total, 1250 articles were screened. Among them, 410 were relevant for this review.

## Results and discussion

### Challenges of PBM in obstetrics

Due to the risk of postpartum haemorrhage (PPH), which is inherent to every pregnancy, PBM is of particular importance for obstetrics. Implementation of PBM in obstetrics provides the opportunity to minimise bleeding and blood transfusions. However, large well-designed clinical studies are missing in this field.

It is more challenging to implement PBM in obstetrics than in other specialties. Furthermore, personnel, commodities, infrastructure, and a universal access to comprehensive obstetric care for the pregnant women are prerequisites [13]. In obstetrics, there are several guidelines for the management of PPH. Most management recommendations are similar; only minor differences exist due to the lack of published solid clinical outcomes. Comparing guidelines for PPH in different obstetrical societies and international groups, there are important differences regarding RBC transfusions and PBM [14].

Challenges of PBM include identification of women at high risk (patient in whom PPH is likely to occur and where RBC transfusion constitutes the standard of care), implementation of medical and surgical/interventional strategies, and restrictive but clinical appropriate blood transfusion in patients who need it. A multidisciplinary approach is prerequisite for the success of PBM in obstetrics, including midwives, obstetricians, anaesthetists, interventional radiologists, and haematologists. Albeit PBM is better described in planned surgery like an elective caesarean section, it should  be applied to any procedure with a certain likelihood of excessive bleeding, including vaginal delivery or non-elective caesarean section. PBM in obstetrics starts during antenatal care or even preconceptionally.

### Haemodynamic changes during pregnancy

In pregnancy, a range of physiological modifications in the haemodynamic, cardiovascular, and coagulation-fibrinolysis systems occur that are designed by nature to prevent blood loss during delivery. During the first trimester, there is an increase in blood volume [15]. The volume of blood continues to expand rapidly during the 2nd trimester (30–50%) before it reaches a stable level in the last 3 months. In parallel, the amount of RBC increases but to a lesser extent (20%), leading to a relative anaemia due to haemodilution [16], which reaches its maximum by 30–32 weeks of pregnancy. Dilutional decrease of haemoglobin is, therefore, a common physiological process in pregnancy, especially between weeks 28 and 34, when haemoglobin concentrations are lowest. In the first months of pregnancy, the red blood cell mass increases about 18–25%, followed by a drop after childbirth due to peripartal haemorrhage [17–19]. The increase in RBC mass ensures enough oxygen for the increased demands from both mother and foetus. These physiological changes have considerable advantages during pregnancy: The placenta has a better perfusion, the risk of thrombosis decreases, and an adequate blood supply is ensured despite the bleeding that occurs with childbirth [20–22]. Uterine artery blood flow increases during pregnancy (10 times) and reaches 450-750 ml/min at term [23].

In parallel, there is a substantial increase in clotting capacity with an increase of the coagulation factors I (fibrinogen), VII, VIII, IX, X, XII and von Willebrand factor. Furthermore, there is a decrease of F XIII and a physiologic decrease of protein S, while F II, V and protein S do not change [24].

The elevation of plasminogen activator inhibitors 1 and 2 diminishes fibrinolytic activity. Thus, there is an increase of the thromboembolic risk. In summary, haemodynamic and haemostatic changes represent adaptations of nature to the challenges of reproduction and are prerequisites for a successful pregnancy outcome of the mother and her child. Nevertheless, PPH remains a main factor of maternal morbidity and death during childbirth [25].

### Causes of postpartum haemorrhage

Main reasons for severe PPH include uterine atony, retained placenta, placenta praevia, placenta accreta, placental abruption, trauma involving uterine rupture, or lower genital tract trauma and primary coagulopathy [26–28]. The causes of PPH from a clinical perspective are best summarized as the 4 T’s: Trauma (of birth canal), Tissue (remaining placenta or placental pieces), Tone (decreased uterine muscular tone: atony), and Thrombin (coagulopathy). Women with previous PPH in the last pregnancy, pre-existing anaemia, prior caesarean section, multiple gestation, uterine fibroma, preeclampsia, obese women, chorioamnionitis, and foetal macrosomia are at increased risk for PPH [29].

However, PPH can occur in every pregnant woman, and most women (61%) with PPH do not have a risk factor excluding maternal age and caesarean section [30]. Therefore, we have to consider that all pregnant women are at a considerable risk for PPH. As a consequence, it is also not possible to define those women who have a very low risk for PPH.

### Iron deficiency and anaemia in pregnancy

Preoperative anaemia is often neglected in surgery. The surgical intervention is often performed as planned, and blood is given when considered necessary (part of the standard care) [12]. In obstetrics, there is a unique opportunity to detect iron deficiency and anaemia a long time before a potential blood loss, as well as in each of the following visits of pregnancy care. Therefore, optimal prerequisites exist to implement the first pillar of PBM, and they essentially include the optimization of the red blood cell mass at delivery. In general, anaemia occurs frequently in pregnant women and is mostly associated with iron deficiency. Other causes include haemoglobinopathies [thalassemia, sickle cell anaemia], infections [hook worm, malaria], Vitamin B_12_ deficiency, or chronic inflammation. In 2011, the percentage of anaemic pregnant women worldwide was 38%, with wide range between different regions: For example, in the Middle East Region, 48.7% of the pregnant women had anaemia, in Africa 46.3%, and in Europe 25.8% [31]. In most cases, it is in theory possible to find the reason for anaemia and to treat it correctly during pregnancy, thereby improving the outcome of mother and child.

Due to the lack of clear data reference, haemoglobin values (Hb) during pregnancy are discussed controversially. According to the WHO, during pregnancy, the diagnosis of anaemia is confirmed if Hb is < 11 g/dL [32]. It is of use to have different Hb reference values in each pregnancy trimester, because it is recognized that between 3 and 6 months of pregnancy Hb-value is reduced by 0.5 g/dL.

The primary reason for anaemia in pregnancy is iron deficiency [31]. Iron need increases considerably during pregnancy. Most of the time, iron stores are insufficient to fulfil this increase, which is the consequence of physiological changes (erythrocytes mass ~ 450 mg, placenta ~ 80 mg, bleeding during vaginal delivery birth ~ 250 mg, and foetus ~ 225 mg). The iron needs during pregnancy are estimated to 1 g of additional iron. If the women breastfeeds her child, she will need an extra 1 mg iron per day [33]. Bone marrow biopsies showed that if no extra iron is provided during pregnancy, 80% of subjects will have exhausted iron reserves at delivery [18]. Of course, the above-mentioned other reasons for anaemia have to be considered and if necessary treated [32–34].

NICE (The English National Institute for Health and Care Excellence) recommends a complete blood count at the beginning of pregnancy and at 28 week gestation, to allow an optimal treatment if anaemia is diagnosed [35]. Other national societies, such as the Swiss Society of Gynaecology and Obstetrics (SSGO), recommend a full blood count and a ferritin value at booking, and thereafter haemoglobin levels every trimester. The reason for low Hb levels should be clarified and supplementation (iron, B_12_, etc.) initiated if needed. If anaemia is diagnosed during pregnancy, the following haematological investigations can elucidate the cause of anaemia: a mean red cell volume (MCV) can support the clarification of the causes of anaemia. It can advocate: (a) microcytic anaemia due to iron deficiency or haemoglobinopathy; (b) macrocytic anaemia related to deficiency in vitamin B_12_ or folate; and (c) normocytic anaemia related to maternal diseases/infections. Of note, in many cases, anaemia in pregnancy is a mixed form of anaemia, such as iron deficiency combined with vitamin B_12_ deficiency, which may render red blood cell indices less reliable as diagnostic feature. Complementary blood tests are often necessary, and include several haematinic parameters (ferritin, vitamin B_12_, and folate). In particular circumstances, to exclude haemolysis and make a final classification of anaemia, additional haematological and/or enzymatic parameters should be performed [36–38]. In cases of microcytic anaemia with normal ferritin, investigation of haemoglobinopathies should be undertaken depending on the origin of the patient.

Iron deficiency can, therefore, not be excluded if the MCV is normal. Haemoglobinopathies should always be suspected in case of severe hypochromic microcytic anaemia. Red cell distribution width (RDW) may help to differentiate iron deficiency from other microcytic anaemias, e.g., in case of haemoglobinopathies. Thus, if mean corpuscular haemoglobin is below 26 pg, it is important to screen for haemoglobinopathies in case of normal iron stores. Regarding iron stores, serum ferritin seems to be the best and most practical marker. In pregnancy, a serum ferritin concentration < 30 µg/L implies insufficient or empty iron stores and, therefore, an increased risk for developing iron deficiency anaemia. A serum ferritin value < 12 µg/L implies established iron deficiency with empty iron stores at all stages of pregnancy [36–38]. As an acute-phase protein, ferritin increases during inflammation or infectious episodes. Therefore, the measurement of the C-reactive protein (CRP) level at the same time is recommended. A normal serum ferritin value does not exclude iron deficiency in inflammatory situations. Because iron deficiency is very common in pregnancy, serum ferritin screening during first trimester is in several most recent recommendations, for example, by the SSGO [40]. If iron stores are empty in early pregnancy, iron treatment is medically indicated even if the haemoglobin is still normal. As iron demand increases during gestation, untreated iron deficiency will result in incremental anaemia. As outlined above, women suffering from anaemia have an increased risk during PPH or even moderate bleeding.

### Prevention and treatment of antepartum iron deficiency and anaemia

To prevent iron deficiency and iron deficiency anaemia, an alimental iron supplementation of 30–60 mg/day is recommended by the WHO [39] for all pregnant women. There is no recommendation neither in the UK nor in Switzerland for routine iron supplementation in all pregnant women [40, 41]. In countries with an increased prevalence of iron deficiency and anaemia, particularly in low resource countries, routine supplementation of iron seems to be an appropriate strategy. However, iron supplementation may cause undesirable side effects such as gastric pain or constipation in up to 25% of pregnant women [42]. Therefore, targeted iron treatment in women with iron deficiency anaemia or low iron stores without anaemia is the preferred strategy in populations in which iron deficiency in pregnant women is not extremely high. Hence, it is recommended to check serum ferritin at the beginning of pregnancy and offer oral iron supplementation if serum ferritin is below 30 ng/ml.

In women with mild-to-moderate iron-deficiency anaemia during the first and second trimesters, it is advised to have a daily oral iron substitution (160–200 mg/day of iron) [40]. This is also applicable for iron deficiency without anaemia in early pregnancy (serum ferritin < 30 ng/mL) knowing that the iron need will increase during gestation [40, 41, 43].

Intravenous iron is recommended if there is intolerance to oral iron preparations (gastrointestinal side effects) if Hb levels do not increase appropriately (less than 1 g/dL within 14 days) due to impaired intestinal absorption or poor compliance, in cases with severe, advanced or progressive anaemia (Hb < 9 g/dL), or if a rapid anaemia treatment is necessary due to advanced gestational age or in Jehovah’s witnesses [32, 40]. The choice of parenteral iron preparation depends on the products availability in different countries. Today, iron dextran products are not recommended due to the increased risk of anaphylactic reactions compared to the newer intravenous iron products available (i.e., ferric carboxymaltose, iron sucrose, iron gluconate, etc.) [44]. Based on the available clinical trial data of iv iron treatment in pregnancy [45–48], in Switzerland, the SSGO recommends ferric carboxymaltose use as best treatment choice when parenteral iron use is appropriate [40].

### Management of the woman during delivery

Risk factors for PPH are older age, multiple gestations, preeclampsia, induction of labour, uterine fibroma, polyhydramnios, placenta praevia, and placenta accreta spectrum disorder. The mode of delivery cannot be considered as a predictor for PPH, although caesarean section is associated with a higher average blood loss as compared to vaginal birth. Recent evidence suggests an association between a low pre-partum haemoglobin and an increased risk of PPH. In an observational study with 53 patients with PPH, 21 women had emergency hysterectomy because of serious uterine atony, whereas the remaining 32 women were responsive to standard of care (conservative measures) [49]. Prepartum haemoglobin value was low (< 7 g/dL) in 81% of women with hysterectomy and PPH (*n* = 17/21) compared to 12.5% of the non-hysterectomised women. These data showed a strong relationship between anaemia (low Hb values < 10 g/dL) and risk of PPH. The rationale behind this may be decrease myometrial contractility and/or impaired coagulation due to low Hb levels.

Among the most current guidelines are those of the German, Austrian, and Swiss Societies for Gynaecology and Obstetrics published in 2018 and the RCOG (Royal College of Obstetricians and Gynaecologists) published in 2017 [50, 51]. Risk factors for PPH are often present before birth, according to retrospective studies in about 40% of women with PPH. In these women, the clinical management during gestation must be modified. One important issue is the facility of birth: Women with a potential of haemorrhage are advised to give birth in a centre, where all required care is onsite.

During delivery, blood loss evaluation is often challenging, but important for clinical management of the patient if bleeding is increased. Typically, blood loss is underestimated by 50% during vaginal delivery. Blood collecting bags or weighing swaps may help for a more accurate estimation of blood loss. The rapid and appropriate management in case of PPH is crucial to reduce morbidity and, in particular, reduce the need of blood transfusions. Clinicians should be prepared and well trained to use timely appropriate medication, as well as mechanical and surgical interventions to stop PPH. The key factor is to perform a rapid but thorough examination to identify the cause of haemorrhage (4TS) and treat it accordingly. The therapy of PPH after vaginal birth or caesarean section depends on the clinical situation and the cause of PPH (4 T’s: Trauma, Tissue, Tone, Thrombin, see above) and consists of different therapeutic measures: Suturing of tissue laceration in birth canal or uterine rupture, surgical removal of placental tissue, uterotonic medications, and administration of Pro-coagulants. If bleeding persists, further measures include insertion of a Balloon-tamponade of the uterus, surgical intervention such as uterine compression sutures (B-Lynch, Hayman technique, or others [52]) or uterine vessel mass ligation. In haemodynamically stable patients, emergent transfer to interventional radiology department and uterine artery embolization may be an effective and often successful intervention [53]. Finally, hysterectomy might become necessary as ultima ratio if other measures were unsuccessful.

### Prevention of PPH

To minimise the risk of PPH, Cochrane reviews [54–56] have shown that the proactive management of the third stage of labour which consists of prophylactic administration of uterotonics such as oxytocine, carbetocin, or ergometrine is successful in preventing PPH. The main component of the package is the use of oxytocin or carbetocin, as controlled cord traction adds only very little [57]. Early cord clamping limits the blood volume to the newborn and may lead to iron deficiency and anaemia in preterm born babies [58]. It has been shown that delaying umbilical cord clamping (minimum 1 min.) have an extended benefit (up to infancy) for the new-born [59]. Therefore, systematic early clamping of the umbilical cord is not advised anymore, except in emergency cases such as severe foetal distress or placental abruption.

### Fluid management

Several physiological changes occur during pregnancy. In the first months, the body of the pregnant woman holds off about 500 up to 900 mEq of sodium [60–62], which increases the body water volume by 6–8 L. Development of resistance to angiotensin II occurs in parallel to an upregulation of the renin-angiotensin system. This leads to a broad rise of 4–7 L in the volume of extracellular water and a reduction of sodium and water elimination to keep a physiologic blood pressure [63, 64]. Stroke volume and heart rate increase. Cardiac output rises rapidly at the beginning of the 5th week of gestation (first trimester) and continues until the 32nd week. It reaches the maximum around the 6th month of pregnancy, having reached ~ 130–150% of the values of non-pregnant women. After birth, cardiac output decreases and turns back to normal levels only at 24 week postpartum. Colloid osmotic pressure (COP) may decrease from 25 mmHg to 18–20 mmHg and cause oedema. All the factors mentioned above should be kept in mind and taken into consideration for the fluid management of complicated obstetric patients.

During severe PPH, decreased cardiac output, hypotension, and vasoconstriction may cause a decrease in end-organ perfusion of vital organs, including kidneys, heart, and brain [65]. Fluid management is a crucial component of PPH treatment to maintain adequate tissue perfusion. Not only blood loss is difficult to estimate, the same is true for volume status of the patient. Due to the physiological increase in blood volume and cardiac output, the parturient may present with cardiovascular stability despite significant blood loss. Shock index (heart rate divided by systolic blood pressure) above 1 is considered a helpful marker to identify relevant hypovolaemia [66]. Maternal lactate is also used by anaesthetists to monitor tissue hypo perfusion in case of significant blood loss. It is important to appropriately warm the infused volume to avoid hypothermia, which has a negative impact on coagulation, oxygenation acidosis [67]. There is no consensus on the choice of crystalloid or colloidal infusion [68] and there is no standard for the appropriate volume of resuscitation fluids [69–71].

### Treatment of PPH 

Uterine atony is the most frequent reason for PPH. Therefore, methods to stimulate contractions of the myometrium should be used. According to Mavrides et al. (a Cochrane review), the management can start at the following sequence [50, 72]: 

1. Uterine fundus palpation and rubbing for stimulation of contractions.
2. Bladder emptying (leave the Foley catheter in place). Ensure a complete placenta or uterine vacuity if doubt.
3. Intravenous injection (i.v.) of Oxytocin 5 IU (additional dose might be needed).
4. Parenteral administration of Oxytocin (40 IU in 500 mL isotonic solution at 125 ml/h) except if there is a need of fluid limitation.
5. If the previous interventions did not lead to appropriate response, sulprostol should be administered by controlled infusion.
6. In case of non-availability of sulprostol, 800 micrograms of misoprostol can be used rectally or other uterotonics including prostaglandin F2alpha or ergometrin may be administered.

Expert consensus supports the use of these methods, even though no evidence-based trial was published [73].

### Tranexamic acid

Antifibrinolytics such as aminomethylbenzoic acid, aprotinin, aminocaproic acid, and tranexamic acid diminish bleeding. They act by the inhibition of fibrin clots breakdown [74, 75]. It has been shown in surgery that tranexamic acid can reduce bleeding and consequently reduce by 1/3 the need for RBC transfusion [76]. Large clinical trials have shown that early administration of tranexamic acid reduced mortality in trauma patients. Similarly, a large multicentre RCT in women with PPH has shown reduction of morbidity as well as mortality. Importantly, the effect was best if tranexamic acid was administered within the three first hours after the onset of the haemorrhage [77–79]. It is of note that in these patients, most severe bleeding and death occurs within 12 h after haemorrhage start. In PPH, death peaked 2–3 h after delivery. The important WOMAN trial has shown that there is an improvement of survival with the use of tranexamic acid [78]; however, it should be used rapidly otherwise the benefit reduces by 10% per 15 min delay up to no benefit reported after 3 h [80]. Of note, the WOMAN trial has been subject to criticism regarding the issues of the included patient population and regarding the number needed to treat. Patients with increased blood loss during vaginal birth or caesarean section should receive antifibrinolytic drugs as soon as possible for the reasons mentioned above. While bleeding, the first coagulation factor to drop to critically low levels is fibrinogen. Therefore, early inhibition of fibrinolysis is reasonable and tranexamic acid must be given before substitution of fibrinogen. Early administration of tranexamic acid is, meanwhile, recommended as the first line of treatment for women with increased blood loss during birth.

In women who were treated prophylactically with tranexamic acid (1 or 0.5 g i.v.) plus the usual uterotonics after vaginal delivery or caesarean section, bleeding beyond 400 or 500 ml was less frequent [81]. A French randomised controlled study comparing 1 g of prophylactic tranexamic acid with placebo showed an effect on the incidence of PPH [82]; however, more data are needed before prophylactic tranexamic acid can be recommended [50].

### Red blood cell transfusion

During PPH, it is of high importance to make the difference between active bleeding and non-active bleeding regarding the indication for RBC transfusion. In principle, during active bleeding in the acute phase of PPH, the dynamic of the clinical situation can be extremely high, and quick RBC transfusions often save the life of the patient. Criteria for transfusion mainly include estimation of blood loss, haemodynamic situation and tissue oxygenation level (lactate), haemoglobin (Hb)/haematocrit (Ht), and prediction of severity of PPH (e.g., by measuring fibrinogen). Measuring just Hb/Ht is not the most reliable way to monitor the progress of the clinical situation. Assessment can be inaccurate leading to a delay of blood transfusion; therefore, further clinical criteria need to be taken into account for an optimal monitoring of the situation, specifically haemodynamic criteria. In haemorrhage and resuscitation, the European Society of Anaesthesiology advocates that Hb/Ht along with base deficit and serum lactate should be measured repeatedly for tissue perfusion and oxygenation evaluation [83]. It should, however, be kept in mind that physiological metabolic compensation of respiratory alkalosis is common in pregnancy, and therefore, a base deficit of up to -3 is physiological. Base deficit with a concomitant increase of serum lactate is a sign of hypoperfusion, usually due to hypovolemia. Correction of hypovolemia with crystalloid fluids might unmask anaemia.

In the later phase of PPH, when bleeding is stopped, active (internal) blood loss is excluded and the haemodynamic stability and compensation of the patient is confirmed, the indication for blood transfusion should be more restrictive with no fixed criteria for RBC transfusions [84, 85]. Hb below 6 g/dL does usually require a RBC transfusion, while this is rarely the case in a haemodynamically stable situation with an Hb of 8 g/dL or above. Between 6 and 8 g/dL, transfusion indication should be more restrictive, depending on the clinical situation and on patient’s symptoms. Often, red blood cell transfusion can be avoided, and instead, intravenous iron treatment can be initiated 8if iron stores have been short) for rapid recovery of Hb levels and alleviation of clinical symptoms.

### Fibrinogen concentrate

Fibrinogen is an acute phase protein and is essential for effective haemostasis. It has been shown that fibrinogen levels measured during PPH correspond well to the severity of PPH, although it is unclear if low fibrinogen values contribute to the pathogenesis of PPH or are a secondary phenomenon of blood loss [86–88]. Several studies [89–96] have suggested that fibrinogen might be useful in normalizing standard laboratory tests in PPH associated with hypofibrinogenaemia. Much of the evidence supporting a benefit of fibrinogen concentrate for PPH is based on case series, retrospective register investigations, or uncontrolled, non-randomised studies [97]. Despite this, early administration of fibrinogen has already been included in most guidelines [98]. For example, the European Society of Anaesthesiology recommends to provide fibrinogen if the levels are < 2 g/dL [83], this is also the case for several obstetric societies like the RCOG [50]. Fibrinogen concentrate is associated with reduced bleeding and a decreased need for transfusions in PPH. Fibrinogen concentrate should be given in severe PPH, particularly if fibrinogen levels are low. Because measurement of fibrinogen levels is time consuming and, therefore, impractical in ongoing PPH, bedside tests, such as thromboelastometry, are recommended. This may enable a rapid correction of coagulopathy due to consumption and dilution [99].

### Transfusion of fresh frozen plasma

Fresh-frozen plasma (FFP) has a limited clotting capacity and is inferior to fibringogen concentrate for the treatment of hypofibrinogenaemia. As a colloidal infusion, FFP is given for volume resuscitation in situations with severe hypovolemia and concomitant coagulopathy. Thawing FFP is time-consuming, and therefore, timely organisation of FFP is recommended [50].

### Recombinant activated human factor VII (rhFVIIa)

Recombinant human factor VIIa (rhFVIIa) is a tissue factor-activated prohaemostatic agent. Even in severe PPH, the systematic use of rhFVVa has not been recommended [49]. Efficacy of rhVIIa has been shown in non-randomised studies in severe PPH. The risk of thromboembolic complications has not been systematically investigated. To be effective, rhVIIa requires correction of hypothermia, acidosis, fibrinogen levels, and anaemia [100]. If haemorrhage could not be controlled by other measures, rhFVIIa could lower the need of second-line therapies [101]. In a prospective cohort study with 22 patients with severe PPH rhVIIa contributed to the control of PPH and hysterectomy was avoided [102]. In life-threatening PPH, rFVIIa administration might be used. However, this should not replace or postpone vital interventions. It is of note that patients should be monitored to detect thromboembolism, especially if rhVIIa is given in the combination with tranexamic acid.

### Surgical/interventional strategies as second-line treatments for PPH

#### Uterine balloon tamponade

It is possible to manage the majority of severe PPH situations when blood loss is rapidly minimised and stopped using appropriate methods, for example, with uterotonics and other interventions. However, these therapies are not always effective in stopping severe bleeding. Therefore, to manage severe PPH and reduce morbidity and mortality, it is necessary to combine several methods including second-line treatments which can be applied even in low-resource structures. In this case, uterine balloon tamponade can be useful. It is a relatively simple and effective approach. A balloon device (Bakri-balloon) is introduced in the uterus and filled with fluid to apply pressure on the uterine walls and allow haemorrhage discontinuation. When this method works to stop bleeding, it is unlikely that the patient needs surgery (in particular, laparotomy after vaginal birth) and RBC transfusions. In the situation, where balloon tamponade is not fully effective to stop severe bleeding, it can help to stabilise the situation until the patient is transferred to a structure which has more treatment options (bridging method). Balloon tamponade works quite fast (5–10 min.) after start of use. Balloon tamponade can help in unmanageable PPH or in areas/structures, where efficient methods for PPH are unavailable. Therefore, it has been recommended in several guidelines (The WHO, the American College of Obstetricians and Gynaecologists, the International Federation of Gynaecology and Obstetrics, the RCOG, and the International Confederation of Midwives) [103–105].

#### Embolization

Arterial embolization after PPH can be successful in arresting the bleeding [106]. Prerequisites for embolization are availability and logistics of interventional radiology, and haemodynamic stability of the patient. In situations, where an interventional radiologist or the logistics to perform arterial occlusion or embolization are not available, uterine balloon tamponade should be considered as first-line treatment. Balloon tamponade can also can be used to bridge the time until embolization. Longitudinal trials in patients who underwent arterial embolization to manage PPH showed that this method does not affect the following menstruation cycles or fertility [53]. If artery ligation failed, selective arterial occlusion might be considered [107]. Embolization seems to work to manage severe postpartum blood loss and to conserve fertility especially in life-threatening situations.

#### Uterine compression sutures

When the relatively non-invasive treatment of Balloon tamponade does not stop PPH, the next step is the use of more invasive treatments requiring laparotomy. If no active bleeding vessel is identified, uterine compression sutures can be applied. In 1997, the B-Lynch uterine compression suture was introduced and became the most widely performed suture. Subsequently, several other uterine compression sutures were described, such as Hayman, Cho, Pereira, Ouahba, or Hackethal suture. Up to date, randomised trials comparing the compression sutures to other procedures are missing. The average rate of haemostasis is 97% with a range from 76% to 100% [108]. The success rate is higher if compression suture is performed within 1 h after delivery. The risk for hysterectomy is increased by fourfolds if there is a 2–6 h delay (after delivery) of uterine compression suture [109].

Ligature of the uterine and utero-ovarian arteries is further surgical interventions and can decrease uterine bleeding by reducing perfusion pressure in the myometrium. It does not harm the uterus and does not appear to impact reproductive function [110]. Iliac artery ligation remains challenging even for an experienced pelvic surgeon [111]. Therefore, arterial embolization, uterine artery ligation, and uterine compression sutures largely replaced this procedure.

#### Cell salvage

Cell salvage is the process of washing and returning intraoperatively collected RBC to the patient. Cell salvage is frequently used in non-obstetric surgeries with anticipated major blood loss. In obstetrics, cell salvage is recommended in individual situations with anticipated high blood loss, such as placenta accreta spectrum disorder, while it is not recommended as a routine procedure for caesarean delivery [112]. Cell salvage is frequently acceptable to Jehova’s witnesses, although individual consent has to be sought.

#### Postpartum hysterectomy

If all treatments mentioned above fail, hysterectomy (HE) is the last resort of PPH treatment. As such, it is also associated with further blood loss. Therefore, the decision for HE should not be delayed too much and decision taken too late when there is haemodynamic instability, severe coagulation deficiency, and further signs of decompensation such as tissue hypoxia or hyperthermia. On the other hand, hysterectomy leads to definitive loss of fertility, reason why it should be used only as last resort treatment. An experienced clinician should decide if and when HE is necessary. The request of a second opinion is advised when possible [113]. Postpartum HE requires an experienced gynaecologic surgeon. It is usually performed as supracervical HE to shorten operation time and decrease additional blood-loss. Only in placenta previa and/or morbidly adherent placenta, it is often necessary to perform a total HE including the cervix.

### Active and non-active bleeding in delivery and postpartum haemorrhage

In general, blood transfusions hold risks. Furthermore, for religious reasons, some women such Jehovah’s witnesses—decline RBC. For these reasons, there must be a clear indication for RBC transfusion, such as risk of ongoing haemorrhage with severe anaemia or cardiac decompensation [114]. A recent paper [114] identified only one small randomised controlled trial with 72 pregnant women investigating the role of proactive (prophylactic) or reactive (restrictive), administration of blood. The conclusion of the authors was not in favour of prophylactic blood transfusion [115]. There are missing studies of the consequences of blood transfusion on the mortality of the mothers or the new-borns. Thus, any endorsement from experts is based on the clinical experience but not evidence based [39]. Severe anaemia during pregnancy was linked to low foetal oxygenation, resulting in foetal morbidity and mortality, and reduction of amniotic fluid volume [116, 117]. In these situations, foetal surveillance and blood transfusion in the mother need to be taken into consideration. [35, 43]. In the case of severe anaemia in pregnancy (e.g., Hb < 7 g/dL), the patient should be addressed to a specialized foeto-maternal medicine care centre. If there is no active bleeding but transfusion deems to be necessary, the minimum amount of blood should be transfused (single-unit). Subsequently, clinical and laboratory evaluation should be performed to reassess the additional need of blood transfusion. Iron stores cannot be replenished by a single RBC concentrate (~ 240 mg iron); thus, the additional administration of parenteral iron is recommended. Importantly, restrictive recommendations for transfusion apply only for patients with stable circulation and no active bleeding. In women with active bleeding, transfusions may not be avoidable in many patients and can safe life. Nevertheless, some women—also if necessary—do not accept blood products, for example Jehovah`s witnesses. In those women, PBM improves both short- and long-term outcomes, due to the synergies between preoperative haematological values optimisation, good volume management, and precise methods of surgery [118], but when these steps fail with active bleeding, the obstetrician has to choose expedite technique to fix the situation i.g. hysterectomy.

### Management of women postpartum

#### Evaluation of postpartum haemoglobin

To determine postpartum haemoglobin might have a great impact on the mother’s well-being. Researchers from Spain found an association between ferritin concentration during the immediate postpartum period and postpartum depression [119]. In women with postpartum depression (PPD), the ferritin concentration was lower and more women had depleted iron stores than those without depression. On average, more patients in the PPD arm compared to the non-PPD arm had depleted iron stores without marginal iron deficiency. The problem with these studies is that ferritin levels postpartum are often erroneously high due to an inflammation in particular after caesarean section and PPH, as ferritin is increased in the acute phase of inflammation. We, therefore, suggest omitting ferritin-level measurements in the postpartum period and instead measure Hb, ideally around 48 h after birth as this usually represents the Hb nadir. Nevertheless, there are increased iron needs during pregnancy and postpartum both directly through to the demands of the developing foetus, and indirectly through breastfeeding depletes iron stores. Iron deficiency in postpartum women may alter neurotransmitter and myelin metabolism and thus increase the risk of PPD in susceptible women. Recently, a Danish study showed a correlation between iron deficiency anaemia and high score on the EPDS (Edinburgh Postpartum Depression Scale). Half of women involved in PPD are undiagnosed and untreated. Appropriate iron treatment may, therefore, decrease the incidence of PPD in postpartum iron deficiency.

#### Postpartum anaemia

Iron deficiency anaemia is very frequent in postpartum and is usually due to postpartum blood loss. It is considered as an important health concern. The SSGO defines postpartum anaemia as an Hb < 12 g/dL within ~ 48 h after delivery (the nadir) [40]. Others define postpartum anaemia as an Hb <11 g/dL 1 week postpartum or Hb <12 g/dL 8 week postpartum [32, 120, 121]. Many women suffer from postpartum anaemia. From 43.807 German women who delivered in 1993–2008, 22% had postpartum anaemia with an Hb <10 g/dL after 24–48 h from birth [122]. In Denmark, 14% of women who received iron suffered from postpartum anaemia 1 week after vaginal birth in comparison with a prevalence of 24% in those who did not receive iron [123]. According to the SSGO, at least one-third up to half of postpartum woman in Switzerland are anaemic [40]. Often, postpartum anaemia is due to prepartum iron-deficiency with or without anaemia and/or acute blood loss during delivery [122, 124, 125]. There are several factors, which could increase the risk of PPH [i.g. ethnicity, inflammation (post-caesarean section), vitamin B12 or folic acid deficiency, multiparity, haemoglobinopathies, and infections] [122, 126–128]. Both postpartum anaemia and iron deficiency can induce several consequences such as reduction of physical and cognitive capacity (fatigue, dyspnoea, etc.), higher susceptibility to infections, disability of breast feeding, higher risk of postpartum depression [122], and compromised ability of bonding to the baby [129]. Postpartum anaemia may also increase health-care costs [114]. Thus, the reasons that could lead to postpartum anaemia should be foreseen and corrected in the best way. The postpartum Hb concentration should be allowed to stabilise after delivery for at least 48 h before a reliable diagnosis of postpartum anaemia is performed. To have a reliable Hb value, one should wait 1 week after delivery [121]. However, if anaemia or iron deficiency is misdiagnosed in immediate postpartum, an estimation of serum ferritin together with a complete blood count (CBC) 4–8 week postpartum is a good alternative [128]. To have an unaltered serum ferritin level, inflammation should be excluded by measuring the CRP [38].

### Prevention and treatment of postpartum anaemia

Prepartum iron deficiency with or without anaemia is closely related to anaemia after birth. As postpartum anaemia can be severe and have durable negative consequences of the mother and her child, postpartum anaemia should be avoided by filling up iron stores during (or even before) pregnancy [130]. According to available studies, oral iron supplementation during pregnancy can lower the risk of anaemia during pregnancy and postpartum [131–133]. In case of asymptomatic mild anaemia, the woman should be treated with oral iron (80–200 mg/day for a minimal duration of 3 months) [40, 130]. The patient should be informed on the correct way of oral iron intake to avoid food interactions and have an optimal absorption [41].

Currently available information suggests that intravenous iron in the postpartum period is beneficial and more efficient than oral iron, particularly in women with moderate or severe anaemia [134, 135]. In the systematic review by Sultan et al., Hb at 6 week postpartum were almost 10 g/dL higher in women with postpartum anaemia who received intravenous iron compared to oral iron. Given the reassuring safety profile of intravenous iron, the weaker Hb response and higher risk of gastrointestinal side-effects with oral iron use, i.v. iron can be considered as a viable treatment option for postpartum iron deficiency anaemia [135].

After birth, treatment of iron deficiency anaemia includes the recommendations according to the SSGO [40]:

- Hb from 9.5 g/dL to 12 g/dL: Oral iron therapy. If oral iron medication is badly tolerated due to gastrointestinal side effects, intravenous iron treatment should be administered.
- Hb < 9.5 g/dL: intravenous iron therapy (as first line therapy, ferric carboxymaltose as first-choice product).

Basis of this recommendation are randomised studies showing a more rapid increase of haemoglobin after intravenous iron as compared to oral iron [45, 46]. In particular, in the postpartum period, a rapid recovery is essential for successful breastfeeding and that the mother is able to cope with all the challenges of care for the neonate.

In case of caesarean section or postpartal inflammation, oral iron may not be efficiently absorbed because of the increased Hepcidin level (iron regulator) [136]. Therefore, in such situations (women with inflammation, severe anaemia or refusing RBC transfusion like Jehovah’s witnesses) parenteral iron should be considered as first-line treatment.

The decision to administer RBC transfusion should be considered according to postpartum Hb value as well as clinical symptoms. It can be appropriate when Hb value is <6 g/dL, or between 7 and 9 g/dL if symptoms of anaemia are severe. As a principle, in postpartum women without active bleeding, transfusion should be restrictive and include administration of only one packed red blood cell, followed by Hb measurement and clinical evaluation to decide if more is necessary. When Hb value is > 9 g/dL, blood transfusion is barely needed [35, 40, 43].

## Conclusion

All pregnant women have a significant risk for PPH. Risk factors do exist; however, 60% of women with PPH do not have a risk factor. Patient blood management in obstetrics must, therefore, not only be focused on women with identified risk factor of PPH, but on all pregnant women [30].

PBM in obstetrics is based on 3 main pillars:

1. Measures during pregnancy.
  Identification of anaemia and its cause, treatment of anaemia, early treatment of iron deficiency, optimization of red blood cell mass in view of delivery.
  Identify patients with a very high risk for PPH (for example placenta praevia, placenta increta, large uterine fibroma, etc.), to appropriately plan logistics, management of delivery and PBM. They should preferably deliver in a tertiary care centre.
2. Measures during delivery.
  Use pre-emptive administration of uterotonics after delivery to prevent PPH.
  Minimize blood loss during delivery using appropriate surgical technique in caesarean section.
  In case of PPH, use appropriate modern measures to minimize blood loss, including balloon tamponade, tranexamic acid and fibrinogen administration, recombinant activated factor VII administration, uterine compression sutures, uterine artery embolization, and cell salvage.
3. Measures during the postpartum phase.
  Manage postpartum anaemia by appropriate iron administration. Optimise the patient’s physiological response to anaemia, treat infections and maximize oxygen delivery to minimize transfusions. Use differential management in active bleeding and non-active bleeding phase of PPH: During active and heavy bleeding, blood transfusions are often necessary, sometimes massive transfusions, to save the life of the patient. However, during non-active bleeding phase unnecessary red blood cell transfusions must be avoided, and a restrictive use of blood transfusion should be applied. In that case, i.v. iron infusion seems a better option than oral supplementation to resolve postpartum anaemia.

These three main pillars should be kept in mind by all professionals taking care of pregnant women, including obstetricians, general practitioners, midwifes, and anaesthesiologists. PBM should be more thoroughly introduced in clinical guidance for pregnancy care and PPH of national and international obstetrical societies.
